# Digital, Rapid, Accurate, and Label-Free Enumeration of Viable Microorganisms Enabled by Custom-Built On-Glass-Slide Culturing Device and Microscopic Scanning

**DOI:** 10.3390/s18113700

**Published:** 2018-10-31

**Authors:** Donghui Song, Haomin Liu, Qiuchen Dong, Zichao Bian, Huixiang Wu, Yu Lei

**Affiliations:** 1 Department of Biomedical Engineering, University of Connecticut, Storrs, CT 06269, USA; donghui.song@uconn.edu (D.S.); qiuchen.dong@uconn.edu (Q.D.); zichao.bian@uconn.edu (Z.B.); 2 Department of Chemical and Biomolecular Engineering, University of Connecticut, Storrs, CT 06269, USA; haomin.liu@uconn.edu; 3 Key Laboratory for Biorheological Science and Technology of Ministry of Education, State and Local Joint Engineering Laboratory for Vascular Implants, Bioengineering College of Chongqing University, Chongqing 400044, China; huixiangwu008@gmail.com

**Keywords:** digital, enumeration, micro-colonies, microscopic scanning, viable microorganism

## Abstract

Accurately measuring the number of viable microorganisms plays an essential role in microbiological studies. Since the conventional agar method of enumerating visible colonies is time-consuming and not accurate, efforts have been made towards overcoming these limitations by counting the invisible micro-colonies. However, none of studies on micro-colony counting was able to save significant time or provide accurate results. Herein, we developed an on-glass-slide cell culture device that enables rapid formation of micro-colonies on a 0.38 mm-thick gel film without suffering from nutrient and oxygen deprivation during bacteria culturing. Employing a phase contrast imaging setup, we achieved rapid microscopic scanning of micro-colonies within a large sample area on the thin film without the need of fluorescent staining. Using *Escherichia coli* (*E. coli*) as a demonstration, our technique was able to shorten the culturing time to within 5 h and automatically enumerate the micro-colonies from the phase contrast images. Moreover, this method delivered more accurate counts than the conventional visible colony counting methods. Due to these advantages, this imaging-based micro-colony enumeration technique provides a new platform for the quantification of viable microorganisms.

## 1. Introduction

In microbiological research, it is essential to accurately enumerate microbial cells. For example, as a key step in developing new antimicrobial agents, the determination of minimum inhibitory concentration (MIC) requires inoculation of a precise number of viable microorganisms [1,2]. To date, the colony-forming unit (CFU) has been used as a gold standard method for estimating the number of viable microbial cells [3]. In this conventional method, liquid microbial suspension is uniformly spread on the surface of semi-solid nutrient agar plate by an inoculation loop. Next, the plate is incubated at an optimal temperature until the cells grow to visible colonies [4,5]. The number of colonies is referred to as a CFU.

The major advantages of agar plate method are that the materials are inexpensive and the procedures are simple enough for trainees to follow. However, long cell culture time (up to a few days) and laborious manual counting result in poor time and cost efficiency [3,6]. To reduce manual work and its related human error, commercial automated colony-counters, such as ProtoCOL^TM^ (Synbiosis, UK), EC2^TM^ (BioMérieux, Marcy-l′Étoile, France), and Scann^TM^ 500 (Interscience, Woburn, MA, USA), have been established through applications of imaging processing algorithms. Nonetheless, the counting results are accurate only when the number of colonies per plate is between 10 and 200 [7]. Thus, serial dilutions of microbial samples are necessary to ensure a countable range. As a major concern of serial dilutions, errors become larger as dilution times increase or total counts per plate decrease [8,9,10].

To address the shortcomings of the conventional method, counting invisible growing micro-colonies (average radius <100 microns) at the very early stage of culturing provides a potentially alternative tool [11,12,13,14]. Specifically, micro-colony counting techniques enable much faster detection of viable microbial cells by significantly shortening cell culture time. These previous attempts can be classified using lensless techniques and conventional light microscopy. Recently, Jung et al. employed a complementary metal-oxide-semiconductor (CMOS) sensor chip to real-time image the microbial cells growing into micro-colonies and demonstrated that micro-colony-based enumeration results were comparable to the conventional method [12]. However, the non-movable small imaging area (5.7 mm × 4.3 mm) and limited sample loading volume (1 μL) failed to exceed the cell capacity of the agar plate. Moreover, limited nutrition and air in their isolated cell culture setup resulted in delayed growth of aerobic bacteria, which would not significantly shorten the detection time. Hence, this on-chip imaging technique seemed not to overcome the limitations faced by the conventional counting method. Compared to the lensless imaging systems, conventional light microscopy can deliver higher resolution of images with clear morphology and contrast of the micro-colonies [15]. Although the field-of-view (FOV) of each image captured by microscope is very small, the microscope is capable of producing a large image in any desired size by stitching numerous images taken from the culture area of interest [16,17,18,19]. In previous studies, wide-field fluorescence microscopy was employed to rapidly enumerate micro-colonies stained by fluorescent probes, which served as viable cell indicators [11,20,21]. However, the counts from the microscope could be 50 times larger than those from the conventional method [11]. In addition to the interference caused by light scattering, lack of solid data of accuracy validation restrained microscopy-based counting techniques from broader applications.

In this study, we demonstrated a novel microscopy-based viable bacteria enumeration technique that employed large-area microscopic scanning to achieve rapid, accurate, and label-free enumeration. Specifically, we first developed a bacteria culturing device for microscopic scanning. This device enabled rapid formation of micro-colonies on a 0.38 mm-thick gel film without suffering from nutrient and oxygen deprivation. Next, an imaging setup was customized for rapid automated scanning of the micro-colonies. As a demonstration, EGFP-expressing *E. coli* was used to evaluate the performance of the bacteria culturing device and the imaging setup. Herein, a modified confocal microscope with a motorized x-y sample holder plane was employed to scan the sample area and create phase contrast and fluorescence images side by side. Using fluorescence as a reference, automatically counting the micro-colonies in the phase contrast image was able to rapidly and accurately provide the number of them.

## 2. Materials and Methods

### 2.1. Materials and Instruments

Luria-Bertani (LB) broth for growing bacteria was purchased from Sigma-Aldrich (St. Louis, MO, USA). Agarose was from Promega (Madison, WI, USA). Silicon wafer was obtained from University Wafer (Boston, MA, USA). Agar, polystyrene petri dishes (diameter 100 mm), glass microscope slides, and coverslips were purchased from Thermo Fisher Scientific (Waltham, MA, USA). *Escherichia coli* (*E. coli*) K-12 expressing enhanced green fluorescent protein (EGFP) was received as gift from Dr. Yongku Cho’s laboratory (University of Connecticut). The fluorescence from *E. coli* expressed EGFP was used only for validation purpose. Images were captured by Nikon A1R confocal microscope (Nikon, Japan) and processed using Nikon NIS-Elements Advanced Research microscope imaging software version 4.40.

### 2.2. Preparation of On-Glass-Slide Bacteria Culturing Device

A microscope glass slide with two pieces of spacers (0.38 mm-thick silicon wafer) on the top of it was placed in a petri dish. Another piece of cover glass slide was then mounted on the silicon wafers to form an empty chamber between the two glass slides. Next, 0.6% (*w*/*v*) agarose were suspended in LB culture media. This mixture was sterilized by autoclaving at 121 °C for 15 min. When the media cooled down below 60 °C, kanamycin was added to a final concentration of 50 μg/mL for the selection of EGFP expression. This molten medium was poured into the petri dish and filled the empty chamber. After the media was kept at room temperature for 20 min and became solidified, the cover glass slide was gently removed to present a thin film of nutrient gel with a flat surface.

### 2.3. Preparation of Bacterial Samples

Since *E. coli* cells carried antibiotic resistant gene as a reporter of the production of EGFP, kanamycin was added to LB broth at a final concentration of 50 μg/mL for the selection of EGFP expression. *E. coli* cells from the stock were inoculated into LB broth and pre-enriched at 200 rpm in a 37 °C shaker for 4 h to ensure the bacterial growth was at log phase. Then, the cells were diluted into various concentrations of interest with phosphate buffered saline (PBS). Bacterial suspension (5 μL) was dropped onto the aforementioned thin film of LB-agarose gel. After the drop completely evaporated, the petri dishes were transferred into a 37 °C incubator. The surface of the culture area was placed upside down to prevent the loss of water content.

### 2.4. Imaging Bacterial Micro-Colonies Using Microscopic Scanning

After 5-h incubation, the glass slide with the thin film was taken out by cutting off the surrounding bulk of LB-agarose gel. Then, the silicon wafer spacers were removed and a glass coverslip was gently mounted on the cell culture area that carries micro-colonies. To ensure the distance between the micro-colonies and the lens constant, the coverslip side of the gel was mounted on a supporting glass slide. This setup was next placed on the motorized x-y sample holder stage of confocal microscope, where the supporting glass slide was at the bottom. After the horizontal plane of micro-colonies was focused by the objective lens, images were continuously captured as the sample holder stage moved along a sequential path programmed by the control panel of Nikon software until the entire sample area was scanned. Herein, tiles of phase contrast and fluorescence images (0.665 mm × 0.665 mm for each tile) were acquired simultaneously by an S Plan Fluor ELWD Ph1 20× objective lens (NA 0.45, Nikon, Japan). Finally, the Nikon NIS Elements software automatically created a large image by stitching all the tiles together.

### 2.5. Image Processing and Micro-Colony Enumeration

The large image obtained from the confocal microscopic scanning was analyzed by using ImageJ software version 1.52 (NIH, Bethesda, MD, USA). First, the threshold was adjusted to enhance the contrast of the objects of interest. Second, the image was binarized to remove the noise by rendering micro-colony regions with clear boundaries as black and surrounding background as white. After the binarization, filling-holes processing was conducted to ensure each closed region represents one intact micro-colony. Finally, micro-colony regions above desired size were outlined and the number of these regions was automatically counted by ImageJ software. 

### 2.6. Bacteria Enumeration Using Conventional Agar Plate

A sterile molten liquid mixture of 2% (*w*/*v*) LB culture media and 1.5% (*w*/*v*) agar supplemented with 50 μg/mL kanamycin was poured into petri dishes. As a gelling reagent, the agar can be replaced by agarose and the concentration of it can be adjusted according to specific needs. After the media was solidified in 20 min, 100 μL of *E. coli* suspension was inoculated onto the surface of the gel by uniformly spreading it using a metal loop. The petri dishes were then incubated at 37 °C. After the bacterial colonies were detectable by naked eyes, the number of colonies was manually counted.

## 3. Results and Discussion

### 3.1. Preparation and Evaluation of On-Glass-Slide Bacteria Culturing Device

**Figure 1:**
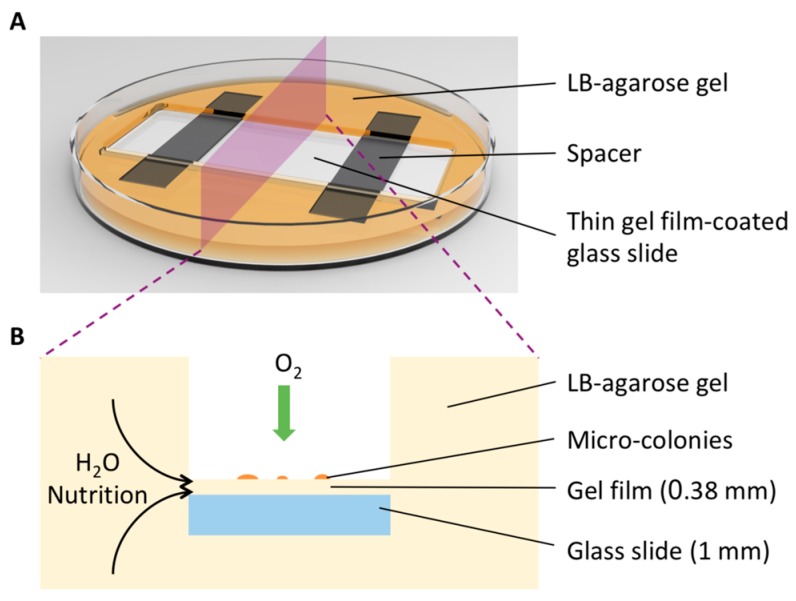
Schematics of the reported custom-built on-glass-slide microbial culturing device. (**A**) A thin Luria-Bertani (LB)-agarose gel film with a flat surface created on a glass slide in a petri dish. The components of the device are described. (**B**) A cross-section of the device showing its capability of culturing microorganisms on the thin gel film without any growth limitation.

In previous studies, micro-colonies were grown and imaged on a film of nutrient gel, which had to be thin enough to deliver good transparency and thereby generate high-quality images. To prevent the thin gel film from drying out, it was sealed in a closed chamber during cell culture and imaging [12,15]. However, limited nutrition and isolating oxygen supplies resulted in slow cell growth. To address this issue, we invented a special on-glass-slide bacteria culturing device (details of preparation are described in the Experimental Section). As illustrated in Figure 1A, a glass slide was embedded in LB-agarose gel. On the top of the glass slide was a flat thin gel film, whose thickness (0.38 mm) was determined by that of the spacer. The cross-section of this device (Figure 1B) shows that the thin film is like a “bridge” connected the bulk gel. This unique design ensures that the film can continuously receive water and nutrition from the bulk gel, as well as oxygen from the ambient air, allowing a long time for incubation without gel drying and rapid formation of micro-colonies without suffering from any restriction on cell growth.

Regarding the composition of the semi-solid culture media, there was only one difference between our system and the conventional method. Here, we used agarose instead of agar to prepare the nutrient gel. Typically, agar is added to aqueous nutrition to form gel matrix, which helps immobilize microbial cells and support them to multiply to colonies. However, the opaque appearance of agar gel determines that it is not a good choice for imaging application [22]. As an alternative, agarose gel has been extensively employed for imaging due to better optical clarity [12,15,23,24,25,26,27,28]. Notably, the flatness of the gel surface is critical for acquirement of high quality scanning image and the accuracy of micro-colonies counting. Hence, we prepared the gel matrix using 0.6% (*w*/*v*) agarose rather than widely used 1.5%, since it was difficult to generate flat thin gel film when the agarose concentration was above 0.6%. Even though the gel becomes softer as agarose content decreases, as previously reported, 0.6% agarose or agar gel still possessed good ability to immobilize microbial cells and support their growth without any issue [24,29].

To evaluate the cell-cultivability of this device, we inoculated the same amounts of bacterial suspension on LB-agarose thin film, conventional LB agar (1.5%) plate, and LB agarose (0.6% and 1.5%) plate respectively and monitored the growth of bacteria. Since inoculation, pictures of these LB agar/agarose plates were captured at 0, 8, 11, 13, 15, and 17 h. It was observed that the bacterial colonies became visible to the naked eye and manually countable at 13 h. The average size of the colonies from these methods showed no remarkable difference (Figure S1), suggesting that the cells on our custom-designed thin gel film grew as fast as on conventional agar plates. Therefore, microscopically analyzing the invisible micro-colonies would significantly shorten the detection time.

### 3.2. Microscopic Scanning Micro-Colonies on a Large Sample Area

Since bacteria cells are transparent in reality and do not affect the amplitude of visible light waves passing through them, high-contrast images are needed for recognizing them. Presented in Figure S2 are images of *E. coli* micro-colonies captured in phase contrast and DIC, respectively. Obviously, compared to DIC, it is much easier to utilize phase contrast to distinguish the micro-colonies from the background. Moreover, a halo surrounding each micro-colony indicates the boundary and further benefits the identification. Therefore, it is more suitable to utilize phase contrast for digitally recognizing the micro-colonies by using image-processing software.

**Figure 2:**
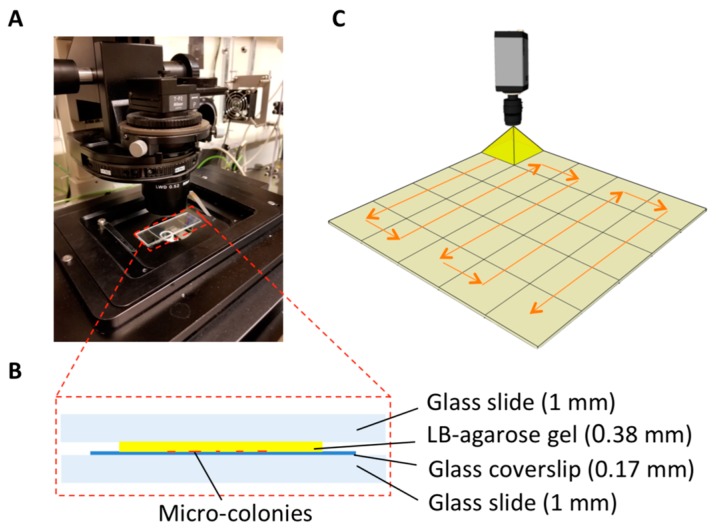
Schematic illustration of the reported micro-colony imaging system. (**A**) a photograph of the experimental setup. (**B**) The components of a sample setup. The thickness of each component is described. (**C**) The scanning of the entire cell culture area on thin LB-agarose gel film is conducted by sequentially capturing images in field-of-views. Each square on the plane represents a field-of-view. The arrows indicate the path of the movement of the camera.

Aiming at recording all the micro-colonies in one image, we employed the microscopic scanning technique to generate an image encompassing the entire sample area. Such scanning was conducted by continuously capturing tiles of images on a certain focal plane and stitching the tiles together into a large image. Considering bacterial micro-colonies growing on gel matrix actually expand in three dimensions [30] and their thickness may not be uniform, significantly more time would be consumed on locating each object through continuously focusing at different depths in the z-direction. To enable fast imaging, we customized the sample setup to ensure that all the micro-colonies could be scanned without conducting any z-scanning. As illustrated in Figure 2B, the setup consisted of a glass slide on the top, a gel film in the middle, a coverslip and a supporting glass slide on the bottom. The micro-colonies at the bottom side of the gel film were compressed on the coverslip by the gravity of the glass slide on the top. The robust flat surface of the supporting glass slide rendered a horizontal coverslip-gel interface, which could be considered as a perfect target plane for the scanning. Moreover, the thickness of the coverslip and supporting glass was fixed, assuring that the distance between the target plane and the lens remained constant. Hence, no concern about any out-of-focus issue was expected.

**Figure 3:**
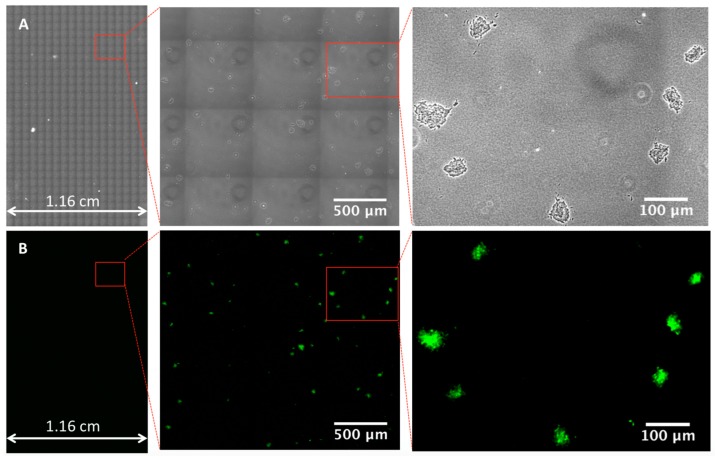
Representative images captured by using the reported platform. Transmitted light (**A**) and fluorescence (**B**) images of bacterial micro-colonies presented on an area of 1.16 cm × 1.81 cm. Fluorescence image was only used for confirmation purpose.

To test the performance of this system, we used EGFP-expressing *E. coli* as a model bacterium and conducted scanning of the micro-colonies grown from them. Although bacteria cells in reality are seldom fluorescent, the green fluorescence from micro-colonies was merely used as a reference to corroborate the accuracy of the result obtained from phase contrast images. Herein, we chose confocal microscopy to meet the specific need of both phase contrast and high-quality fluorescence images in this study. As the LB-agarose gel is a thick specimen with considerable autofluorescence, the confocal microscope can block the out-of-focus light above and below the focal plane by the spatial pinhole and therefore provides higher resolution and enhanced contrast by increasing signal-to-noise ratio [31,32,33]. Additionally, since the transmitted light mode of confocal microscopy can also view the specimen through phase contrast [34], confocal microscopy is capable of simultaneously generating high-quality fluorescence and phase contrast images and viewing them side by side, whereas this cannot be achieved by other microscopy. However, it is worth noting that confocal microscopy is not required for real applications if only phase contrast is employed in enumeration of viable microorganisms. After the bacteria cells on thin gel film were incubated at 37 °C for 5 h, the culture area was scanned at a speed of approximately 17.7 mm^2^/min by a 20× objective lens. Figure 3 displays a large image (1.16 cm × 1.81 cm) created by stitching tiles of images together. The area was large enough to accommodate all the micro-colonies. Zooming in the large image enabled us to identify each micro-colony by phase contrast or fluorescence. It was clear that the micro-colonies presented on the boundaries between the tiles were in their normal shape, suggesting that there was no stitching artifact, such as duplication or disappearance. Therefore, the large image acquisition function presented here was able to accurately deliver the number of the micro-colonies.

### 3.3. Micro-Colony Enumeration

**Figure 4:**
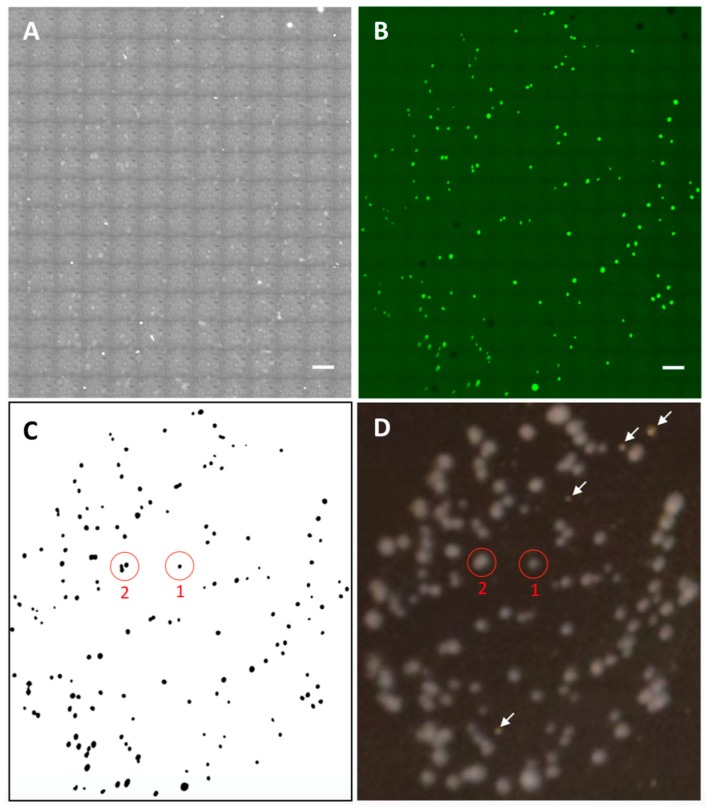
Phase contrast (**A**) and fluorescence (**B**) images of an area (7.9 mm × 9.0 mm) containing micro-colonies grown from the inoculated cells in 5 μL bacterial suspension. Scale bars: 500 μm. Fluorescence image was only used for confirmation purpose. (**C**) An image processed from the raw image in (**A**). The black spots indicate micro-colonies. (**D**) A photograph of visible colonies in situ developed from the micro-colonies shown in (**A**,**B**). The whitish dots were bacterial colonies. The objects pointed by the white arrows were debris, which can be distinguished from the bacterial colonies by the colors. The difference in color can be more easily identified from the high-resolution photograph shown in Figure S3.

**Figure 5:**
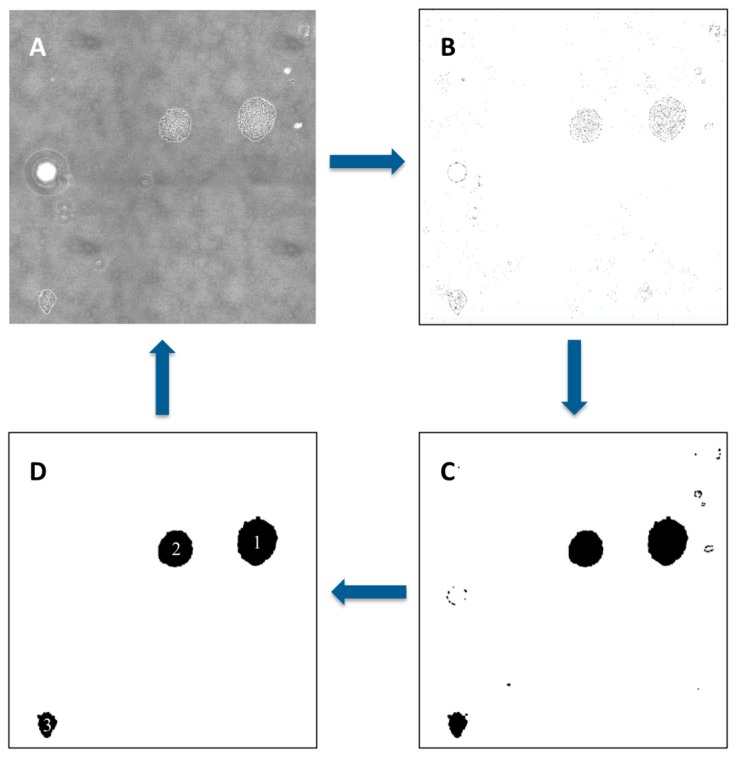
An example of automated enumeration of micro-colonies. The original transmitted light image (**A**) was processed through adjusting threshold (**B**), finding edges, binarization, and filling the holes (**C**), and the black regions above certain size were counted by using ImageJ (**D**).

After the images were acquired by confocal microscopy scanning, the micro-colonies appeared there were enumerated by using image-processing software. Figure 4A presents a selected area containing micro-colonies grown from the cells in 5 μL bacterial suspension. In addition to micro-colonies, empty regions that could be produced by artifacts or tiny air bubbles were also shown in the image. Nonetheless, they do not cause any interference in recognizing the micro-colonies. It is known that phase contrast is generated by the difference in density. Since no other elements, such as the empty regions, throughout the imaged area were as dense as the micro-colonies, micro-colonies can be easily distinguished by filtering the low-contrast objects. Figure 4B shows the corresponding processed image, in which the total number of micro-colonies (dark regions) was counted as 155 (details of the algorithms applied in automated enumeration are described in Figure 5). The locations of the micro-colonies and the number of them were consistent with those obtained from the fluorescence image (Figure 4C), indicating that the result obtained from phase contrast image was trustworthy. To validate if this result represented actively growing micro-colonies, the imaged sample was placed back into the agar plate and further cultured in situ for 20 h at room temperature until visible colonies appeared. The image of these visible colonies (Figure 4D), which was captured by a Nikon D5300 DSLR camera (Nikon, Japan), was used to compare with that of micro-colonies side by side.

Apparently, all the micro-colonies were capable of actively growing, indicating that the number of micro-colonies certainly represents that of viable microbial microorganisms. Furthermore, we found that, multiple micro-colonies in close proximity to each other, which could eventually develop to one single visible colony under the conventional agar plate enumerating method, can be identified and differentiated microscopically using our method. For instance, colony 1 was derived from one micro-colony, whereas colony 2 was developed from three neighboring micro-colonies. This finding suggests that, especially when the density of cells inoculated on an agar plate is high, the counts obtained from the conventional method are much likely to be underestimated. By contrast, our digital enumeration technique can differentiate the neighboring micro-colonies at the earliest stage and be more accurate than counting large visible colonies used in conventional methods. According to specific needs, the upper counting limit can be increased by simply expanding the scanning area. This feature allows that fewer or even no dilutions are needed using our enumerating method, which could reduce the serial dilution-induced error. Furthermore, enumerating around 200 micro-colonies within an area of 60 mm^2^, which is the maximum limit of a 100 mm agar plate, took only around 3 min, freeing up more space and workload on sample preparation through miniaturizing the work on a piece of glass slide. It is worth noting that the scanning time was limited by the function of confocal microscopy. In this study, confocal microscopy was only used to evaluate the performance of our custom-designed bacteria culturing and imaging system in the compatibility with the microscopic scanning function. In practice, since the technique reported here can accurately deliver the counts of micro-colonies only from phase contrast images (fluorescence imaging is not necessary), some recently emerged ultrafast high-resolution microscopic scanning techniques [35,36,37], which can conduct whole glass slide scanning in a few minutes, would be able to further reduce the scanning time per unit area and thereby provide counting result in a timely and high-throughput manner. Overall, the developed technique not only shortened the culturing time, but also allowed the labor-intensive plate counting work to be automatically conducted on a glass slide, thoroughly overcoming the limitations of the conventional method.

## 4. Conclusions

We demonstrated a unique technique that enabled digital, rapid, accurate, label-free enumeration of viable microorganisms using optical scanning microscopy. A custom-built bacteria culturing and imaging system was applied to ensure that all the micro-colonies on the large sample area could be displayed on an image generated by microscopic scanning. Since the micro-colonies can be counted based on their phase contrast rather than fluorescent staining, the procedures are much simplified, rendering this technique applicable to a wide range of microorganisms that can form micro-colonies with clear edges. Employing *E. coli* as a model bacterium, our method reduced the cell culture time from at least 13 h required for the conventional method to within 5 h. Compared to conventional enumerating methods requiring an overnight (18–24 h) incubation, such improvement would benefit the analytical methods that require rapid and accurate quantitative determination of bacteria. Moreover, since the employment of confocal microscopy proved that this enumeration system was compatible with the microscopic scanning function, the application of cost-effective ultrafast microscopic scanning techniques in the future would significantly enhance the performance of our system by miniaturizing the bacteria enumeration work with hundreds of agar plates into a piece of microscope glass slide. Notably, regarding the quantification of viable bacteria, this method is more accurate than the conventional agar plate method due to the fact that neighboring bacteria before merging into one large visible colony can be differentiated using our method. Therefore, this microscopic scanning-based micro-colony enumeration system has great potential for being adopted as a standard tool for quantifying viable microorganisms.

## Supplementary Materials

The following are available online at http://www.mdpi.com/1424-8220/18/11/3700/s1.

## References

[1] Wiegand I., Hilpert K., Hancock R.E. (2008). Agar and broth dilution methods to determine the minimal inhibitory concentration (MIC) of antimicrobial substances. Nat. Protoc..

[2] Balouiri M., Sadiki M., Ibnsouda S.K. (2016). Methods for in vitro evaluating antimicrobial activity: A review. J. Pharm. Anal..

[3] Hazan R., Que Y.A., Maura D., Rahme L.G. (2012). A method for high throughput determination of viable bacteria cell counts in 96-well plates. BMC Microbiol..

[4] Austin B. (2017). The value of cultures to modern microbiology. Antonie Van Leeuwenhoek.

[5] Messer J.W., Rice E.W., Johnson C.H. (2000). Total viable counts. Spread plate technique. Encycl. Food Microbiol..

[6] Davis C. (2014). Enumeration of probiotic strains: Review of culture-dependent and alternative techniques to quantify viable bacteria. J. Microbiol. Methods.

[7] Frost H.R., Tsoi S.K., Baker C.A., Laho D., Sanderson-Smith M.L., Steer A.C., Smeesters P.R. (2016). Validation of an automated colony counting system for group A Streptococcus. BMC Res. Notes.

[8] Sutton S. (2011). Accuracy of plate counts. J. Valid. Technol..

[9] Jennison M.W., Wadsworth G.P. (1940). Evaluation of the Errors Involved in Estimating Bacterial Numbers by the Plating Method. J. Bacteriol..

[10] Snyder T.L. (1947). The Relative Errors of Bacteriological Plate Counting Methods. J. Bacteriol..

[11] Wang X., Yamaguchi N., Someya T., Nasu M. (2007). Rapid and automated enumeration of viable bacteria in compost using a micro-colony auto counting system. J. Microbiol. Methods.

[12] Jung J.H., Lee J.E. (2016). Real-time bacterial microcolony counting using on-chip microscopy. Sci. Rep..

[13] Frost W.D. (1921). Improved Technic for the Micro or Little Plate Method of Counting Bacteria in Milk. J. Infect. Dis..

[14] Jiang C., Chen P., Shan S. (1995). Total microcolony counting on the moving narrow culture band. J. Microbiol. Methods.

[15] Maeda Y., Dobashi H., Sugiyama Y., Saeki T., Lim T.K., Harada M., Matsunaga T., Yoshino T., Tanaka T. (2017). Colony fingerprint for discrimination of microbial species based on lensless imaging of microcolonies. PLoS ONE.

[16] Preibisch S., Saalfeld S., Tomancak P. (2009). Globally optimal stitching of tiled 3D microscopic image acquisitions. Bioinformatics.

[17] Legesse F.B., Chernavskaia O., Heuke S., Bocklitz T., Meyer T., Popp J., Heintzmann R. (2015). Seamless stitching of tile scan microscope images. J. Microsc..

[18] Yang F., Deng Z.S., Fan Q.H. (2013). A method for fast automated microscope image stitching. Micron.

[19] Ma B., Zimmermann T., Rohde M., Winkelbach S., He F., Lindenmaier W., Dittmar K.E. (2007). Use of Autostitch for automatic stitching of microscope images. Micron.

[20] Rodrigues U.M., Kroll R.G. (1988). Rapid selective enumeration of bacteria in foods using a microcolony epifluorescence microscopy technique. J. Appl. Bacteriol..

[21] Baumstummler A., Chollet R., Meder H., Olivieri F., Rouillon S., Waiche G., Ribault S. (2011). Development of a nondestructive fluorescence-based enzymatic staining of microcolonies for enumerating bacterial contamination in filterable products. J. Appl. Microbiol..

[22] Jaeger P.A., McElfresh C., Wong L.R., Ideker T. (2015). Beyond Agar: Gel Substrates with Improved Optical Clarity and Drug Efficiency and Reduced Autofluorescence for Microbial Growth Experiments. Appl. Environ. Microbiol..

[23] Chen M.T., Weiss R. (2005). Artificial cell-cell communication in yeast *Saccharomyces cerevisiae* using signaling elements from *Arabidopsis thaliana*. Nat. Biotechnol..

[24] Gu Y.H., Ko W.H. (1997). Water agarose medium for studying factors affecting germination of conidia of *Ampelomyces quisqualis*. Mycol. Res..

[25] Choi J., Kang J.S., Hong S.C., Bae G.N., Jung J.H. (2017). A new method for the real-time quantification of airborne biological particles using a coupled inertial aerosol system with in situ fluorescence imaging. Sens. Actuators B Chem..

[26] Hong W., Karanja C.W., Abutaleb N.S., Younis W., Zhang X., Seleem M.N., Cheng J.X. (2018). Antibiotic Susceptibility Determination within One Cell Cycle at Single-Bacterium Level by Stimulated Raman Metabolic Imaging. Anal. Chem..

[27] Karanja C.W., Hong W., Younis W., Eldesouky H.E., Seleem M.N., Cheng J.X. (2017). Stimulated Raman Imaging Reveals Aberrant Lipogenesis as a Metabolic Marker for Azole-Resistant Candida albicans. Anal. Chem..

[28] Choi J., Jung Y.G., Kim J., Kim S., Jung Y., Na H., Kwon S. (2013). Rapid antibiotic susceptibility testing by tracking single cell growth in a microfluidic agarose channel system. Lab Chip.

[29] Mitchell A.J., Wimpenny J.W. (1997). The effects of agar concentration on the growth and morphology of submerged colonies of motile and non-motile bacteria. J. Appl. Microbiol..

[30] Su P.T., Liao C.T., Roan J.R., Wang S.H., Chiou A., Syu W.J. (2012). Bacterial colony from two-dimensional division to three-dimensional development. PLoS ONE.

[31] Jonkman J., Brown C.M. (2015). Any Way You Slice It—A Comparison of Confocal Microscopy Techniques. J. Biomol. Tech..

[32] Singh A., Gopinathan K. (1998). Confocal microscopy: A powerful technique for biological research. Curr. Sci..

[33] Wright S.J., Centonze V.E., Stricker S.A., DeVries P.J., Paddock S.W., Schatten G., Matsumoto B. (1993). Chapter 1 Introduction to Confocal Microscopy and Three-Dimensional Reconstruction. Methods in Cell Biology.

[34] Shotton D., White N. (1989). Confocal scanning microscopy: Three-dimensional biological imaging. Trends Biochem. Sci..

[35] Guo K., Liao J., Bian Z., Heng X., Zheng G. (2015). InstantScope: A low-cost whole slide imaging system with instant focal plane detection. Biomed. Opt. Express.

[36] Liao J., Wang Z., Zhang Z., Bian Z., Guo K., Nambiar A., Jiang Y., Jiang S., Zhong J., Choma M. (2018). Dual light-emitting diode-based multichannel microscopy for whole-slide multiplane, multispectral and phase imaging. J. Biophotonics.

[37] Liao J., Jiang S., Zhang Z., Guo K., Bian Z., Jiang Y., Zhong J., Zheng G. (2018). Terapixel hyperspectral whole-slide imaging via slit-array detection and projection. J. Biomed. Opt..

