# Supplementary material for: Digital, Rapid, Accurate, and Label-Free Enumeration of Viable Microorganisms Enabled by Custom-Built On-Glass-Slide Culturing Device and Microscopic Scanning
Source: Sensors (Basel). 2018 Oct 31;18(11):3700. doi: 10.3390/s18113700 (PMC6263752; doi:10.3390/s18113700)
Supplement: Supplementary file 1 [file sensors-18-03700-s001.pdf]

Supplementary Materials

Digital, Rapid, Accurate, and Label-Free Enumeration of Viable Microorganisms Enabled by Custom-Built On-Glass-Slide Culturing Device and Microscopic Scanning

Donghui Song [1] Haomin Liu [2] Qiuchen Dong [1] Zichao Bian [1] Huixiang Wu [3] and Yu Lei [1,2,*]

[1] Department of Biomedical Engineering, University of Connecticut, Storrs, CT 06269, USA
[2] Department of Chemical and Biomolecular Engineering, University of Connecticut, Storrs, CT 06269, USA
[3] Key Laboratory for Biorheological Science and Technology of Ministry of Education, State and Local Joint Engineering Laboratory for Vascular Implants, Bioengineering College of Chongqing University, Chongqing 400044, China
* Correspondence: yu.lei@uconn.edu; Tel.: +1-860-486-4554; Fax: +1-860-486-2959

Contents

Figure S1. The evaluation of the cell-culturability of the custom-built device.

Figure S2. Representative images of bacterial micro-colonies in DIC and phase contrast.

Figure S3. The original photograph of visible colonies shown in Figure 4D.

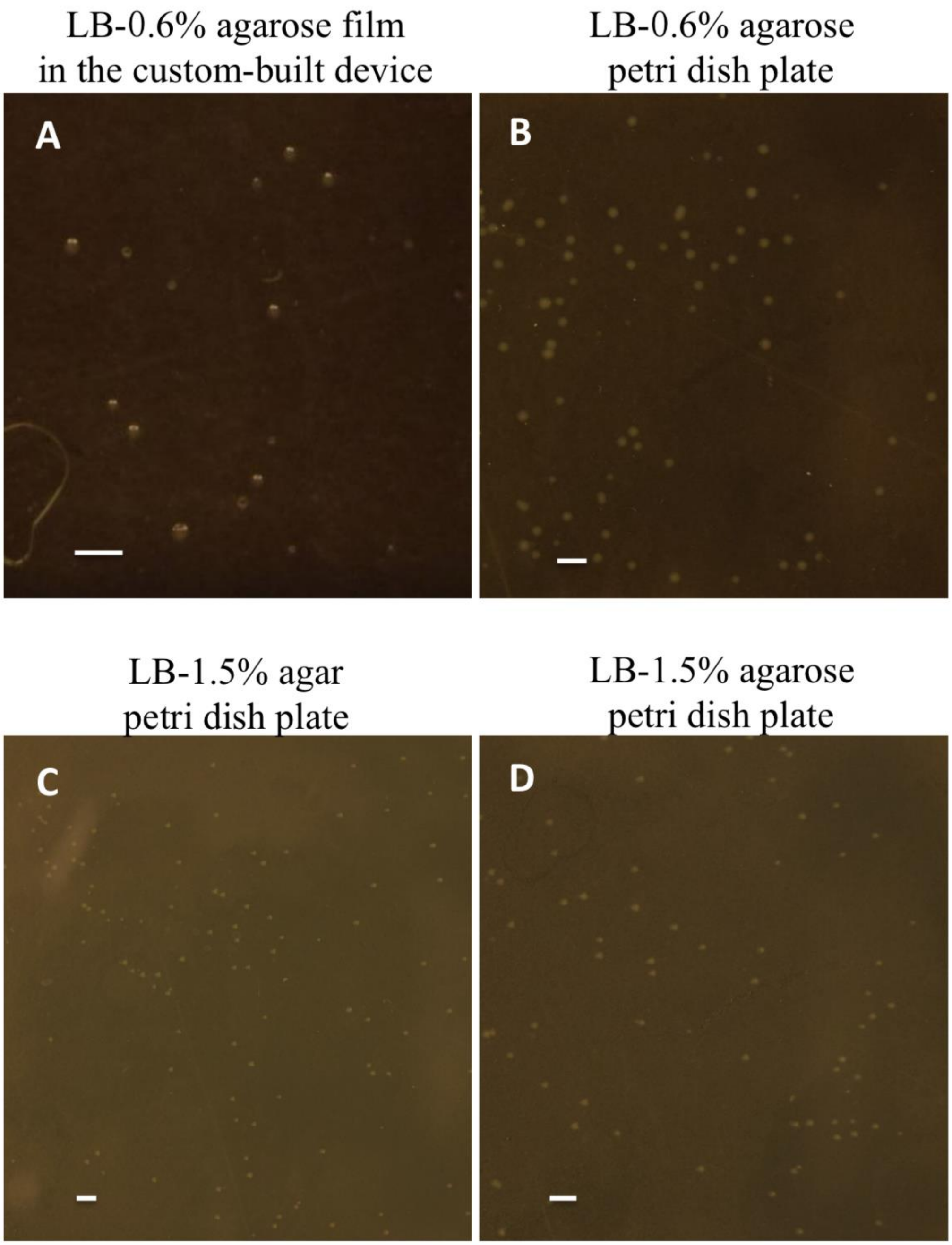

Figure S1. Representative images of bacterial colonies on LB-0.6% agarose thin film in the custom-built device (A), LB-0.6% agarose petri dish plate (B), LB-1.5% agar petri dish plate (C), and LB-1.5% agarose petri dish plate (D). The images were captured after 13 hours incubation at 37 °C. Scale bars: 2 mm.

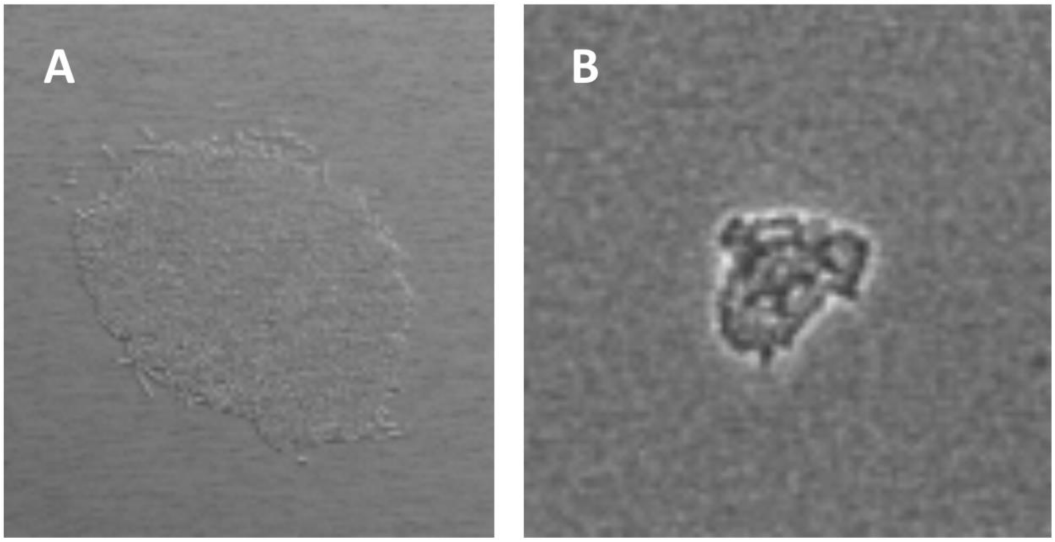

Figure S2. Representative images of bacterial micro-colonies in DIC (A) and phase contrast (B).

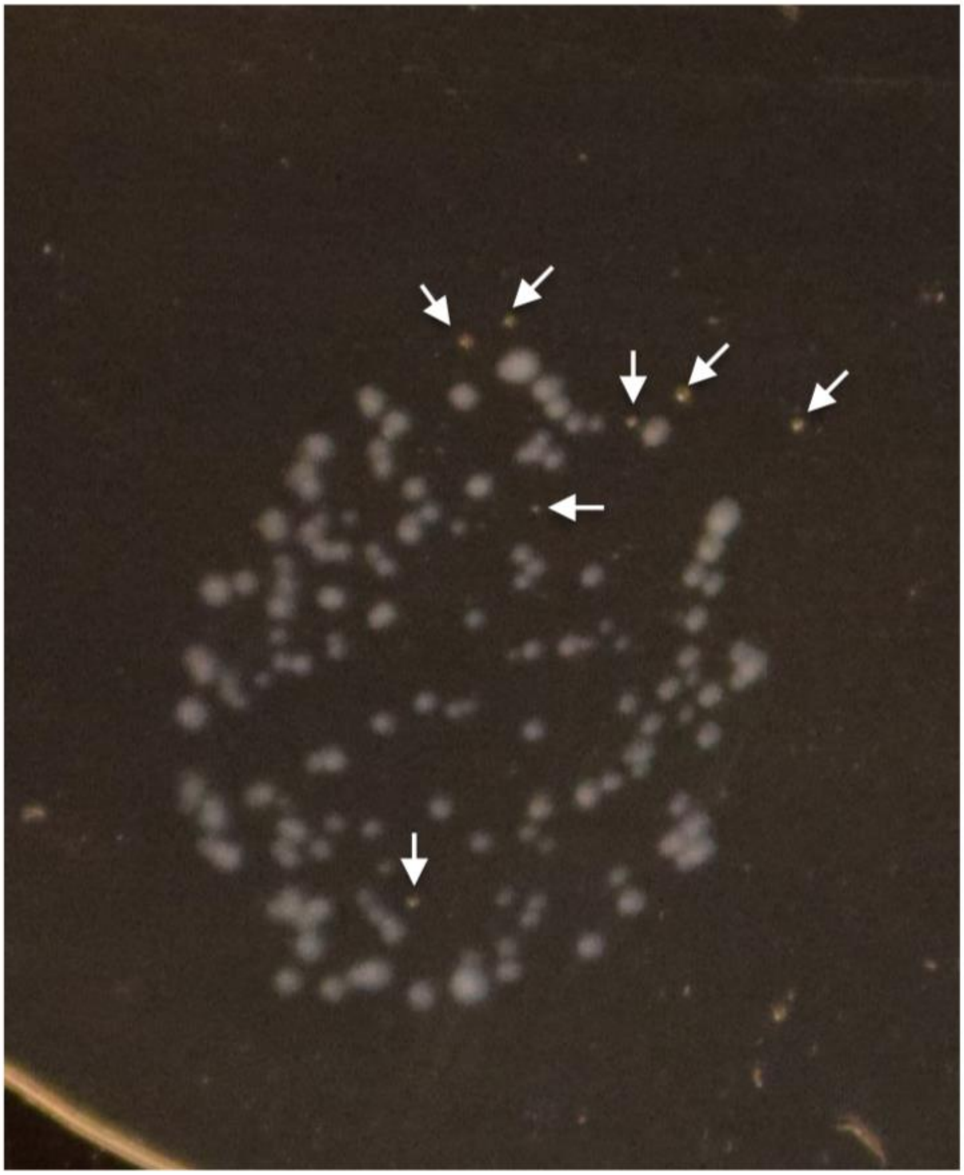

Figure S3. The original photograph of visible colonies shown in Figure 4D. The whitish dots were bacterial colonies. The objects pointed by white arrows were debris, which can be distinguished from the bacterial colonies by the colors.
